# Amplification of the Signal Intensity of Fluorescence-Based Fiber-Optic Biosensors Using a Fabry-Perot Resonator Structure

**DOI:** 10.3390/s150203565

**Published:** 2015-02-04

**Authors:** Meng-Chang Hsieh, Yi-Hsin Chiu, Sheng-Fu Lin, Jenq-Yang Chang, Chia-Ou Chang, Huihua Kenny Chiang

**Affiliations:** 1 Institute of Applied Mechanics, National Taiwan University, 1 Sec.4 Roosevelt Road, Taipei 10617, Taiwan; E-Mails: jang1985@msn.com (M.-C.H.); changco@iam.ntu.edu.tw (C.-O.C.); 2 Institute of Biomedical Engineering, National Yang-Ming University, No.155, Sec.2 Linong Street, Taipei 11221, Taiwan; E-Mail: yihsinchiu07@gmail.com; 3 Department of Optics and Photonics, National Central University, Jhongli 32001, Taiwan; E-Mails: 982406004@cc.ncu.edu.tw (S.-F.L.); jychang@dop.ncu.edu.tw (J.-Y.C.)

**Keywords:** fiber-optic biosensor (FOBS), biosensor structure, evanescent wave, resonator, fluorescent intensity

## Abstract

Fluorescent biosensors have been widely used in biomedical applications. To amplify the intensity of fluorescence signals, this study developed a novel structure for an evanescent wave fiber-optic biosensor by using a Fabry-Perot resonator structure. An excitation light was coupled into the optical fiber through a laser-drilled hole on the proximal end of the resonator. After entering the resonator, the excitation light was reflected back and forth inside the resonator, thereby amplifying the intensity of the light in the fiber. Subsequently, the light was used to excite the fluorescent molecules in the reactive region of the sensor. The experimental results showed that the biosensor signal was amplified eight-fold when the resonator reflector was formed using a 92% reflective coating. Furthermore, in a simulation, the biosensor signal could be amplified 20-fold by using a 99% reflector.

## 1. Introduction

Fiber-optic biosensors (FOBSs) have been widely applied in procedures such as environmental monitoring and biomolecular sensing [1,2]. Researchers have developed various types of biosensors, including ion-sensitive field-effect transistors [3], electrochemistry biosensors [4], quartz crystal microbalance biosensors [5–7], and FOBSs [8]. In particular, fiber-optic sensing is a highly applicable method because it offers several advantages, such as real-time sensing, a compact size, freedom from electromagnetic interference, high sensitivity, and versatility in application [9]. Despite these advantages, its signal-to-noise ratio can be improved [10,11]. An evanescent wave FOBS is an affinity-based optical sensor that leverages an evanescent wave to locally excite targeted biomolecules on the surface of the fiber-optic sensing region. Numerous evanescent wave biosensors have been developed using bioaffinity assays for biomolecule detection, such as DNA hybridization assays [12], immunoassays [13], and surface plasmon resonance biosensor assays [14].

Currently, improving the sensitivity of evanescent wave FOBSs is a critical research direction. Specifically, one problem is that an overly small proportion of the evanescent wave penetrates the core surface of the fiber sensor; when a targeted biomolecule is measured at a low concentration, the entire fluorescent signal cannot be detected. Another problem is that the excitation light passes through the fiber-sensing area only once; thus, the remaining excitation light is wasted. Furthermore, fiber Bragg grating can reflect specific wavelengths of light [15]. For example, it reflects the excitation light, which further passes through the fiber-sensing area, thereby increasing the strength of the evanescent wave. However, fiber Bragg gratings are difficult to produce. On the other hand, Andrade *et al.* presented an evanescent wave optical fiber tip biosensor [16], indicating that the fiber end-face could be coated with a reflective film to prevent the propagation of the light into the bulk solution; however, only one reflective film was contained in the structure, and did not operate in a resonator structure. This study proposes a solution to the aforementioned problems. Accordingly, an experiment and simulation were conducted to demonstrate the novel FOBS resonating structure that fully uses the power of the excitation light.

## 2. Materials and Methods

### 2.1. Reagents and Solutions

A primary antibody (antirabbit IgG produced in goat), an antigen (rabbit IgG), phosphate-buffered saline (PBS), 3-aminopropyl triethoxysilane (APTES), tris(hydroxymethyl)aminomethane hydrochloride (Tris–HCl), sodium azide (NaN_3_), and bovine serum albumin (BSA) were obtained from Sigma-Aldrich (St. Louis, MO, USA); hydrogen peroxide (H_2_O_2_), glutaraldehyde (GA), and sulfuric acid (H_2_SO_4_) were obtained from Fluka (Buchs, Switzerland); and Alexa Fluor 488 goat antirabbit IgG (AF488 IgG) was obtained from Invitrogen (Carlsbad, CA, USA).

The blocking solution was composed of 1% (m/v) sucrose, 0.05% (v/v) tween-20, 0.05% (m/v) NaN3, and 1% (m/v) BSA in the Tris–HCl buffer (50 mM pH 7.4). The GA solution comprised 25% GA mixed with PBS (10 mM pH 7.4) at a ratio of 1:20 (v/v).

### 2.2. Basic Principle and Experimental Setup

**Figure 1.**
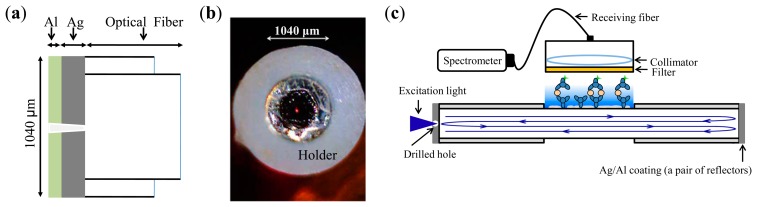
(**a**) Sensor's end-face coated with silver (Ag; 500 nm) and aluminum (Al; 100 nm) to form a reflector; (**b**) 30 μm hole (marked by a red laser-pointer light), at the center of the first reflector, drilled with a 532-nm Nd:YAG laser; (**c**) The biosensor with reflective coatings of Ag/Al on the sensor's end-faces form a resonator structure.

The proximal (near the side with the excitation light source) and distal ends of the FOBS (the optical fiber was a 600-μm-diameter core multimode fiber JTFLH6006301040, numerical aperture (N.A.): 0.37 ± 0.02, 30 dB/km at 488 nm, Polymicro Technologies, Phoenix, AZ, USA) were coated with a 500-nm silver (Ag) layer as a reflective layer [17] and capped with 100-nm of aluminum (Al) to protect the reflective layer, as shown in Figure 1a. A 30-μm-diameter hole was laser-drilled into the proximal reflector, as shown in Figure 1b. The excitation light (488-nm) was coupled through the hole by using a 0.4-N.A. (20×) microscope objective lens. In the reactive (sensing) region of the fiber, the evanescent wave of the guided modes extends from the core layer into the reactive region, in Figure 1c. The evanescent wave in the reactive region represented a small portion of the excitation light. The excitation light was reflected back and forth between the pair of reflectors (*i.e.*, the end faces of the FOBS coated with Ag and Al), which was operating as a Fabry-Perot optical resonator. The back and forth reflections of the excitation light enabled the resonator type biosensor to maximize the use of the excitation light for the excitation of the bonded fluorescent molecules in the reactive region. The evanescent wave excited the fluorescent molecules near the fiber-optic core surface in the reactive region of the biosensor, penetrating approximately 200-nm into the reactive region, in Figure 1c.

Figure 1c illustrates the setup of the fiber optical resonator structure. A 488-nm solid laser (Blue Solid-State Laser, Melles Griot, Carlsbad, CA, USA) was employed as the excitation light source, and a multimode fiber with a 600-μm-diameter core was used as the medium for the biosensor fiber. In fluorescent measurement, a fluorescence-labeled secondary antibody (AF488, Invitrogen) was excited by the excitation light (488-nm), and the peak of the fluorescence emission spectrum was at approximately 520-nm. The fluorescent light was collected by a receiving probe which was composed of a long pass filter (504.7-nm long-pass filter, Semrock, New York, NY, USA), a collimating lens, and a receiving optical fiber. The long-pass filter was used to separate the excitation light and fluorscent light, and the fluorescent light was coupled to the receiving fiber. Finally, a fiber-optic spectrometer (USB 2000, Ocean Optics, Winter Park, FL, USA) was used to measure the fluorescence intensity of the biosensor.

### 2.3. Immunoassay

In this experiment, the cladding layer of the reactive (sensing) region was removed by using a CO_2_ laser and then cleaned with de-ionized water. After the CO_2_ laser removed the cladding layer, a scanning-electron-microscope was employed to observe the cladding removal and establish an experiment standard process. The reactive region was immersed in a 98% H_2_SO_4_ and 30% H_2_O_2_ solution at a volume ratio of 9:1 for 24 h to activate the reactive region [18] for generating the -OH functional groups on the core surface. After the -OH functional groups were generated on the core surface, the reactive region was soaked in a 2% APTES solution for 4 h at 75 °C to form the -NH_2_ functional terminal group, which can immobilize biomolecules [19,20]. Subsequently, the fiber was soaked into the GA solution for 1 h [21] and then rinsed with purified water.

**Figure 2.**
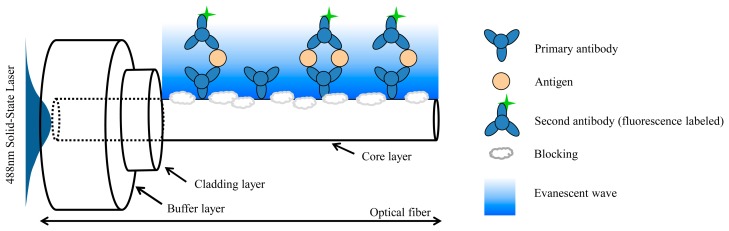
Schematic diagram of a sandwiched immunoassay performed using optical fiber**s** under evanescent wave excitation.

In this study, the FOBS structure was based on a sandwiched biomolecular complex comprising the primary antibody, antigen, and secondary antibody with a fluorescent label, as shown in Figure 2 [22]. The primary antibody (goat antirabbit IgG) was first immobilized on the reactive region of the fiber core surface. Next, to avoid nonspecific binding (*i.e.*, the evanescent wave may excite the nonspecific fluorescent-labeled molecules of the secondary antibody), the reactive region of the optical fibers was immersed in the blocking solution. Subsequently, the targeted biomolecules (antigens) were added to the reactive chamber for 15 min, and the antigen was prepared at a concentration of 500 ng/mL. The secondary antibody with a concentration of 6.64 μg/mL (AF488 IgG) was then added to the reactive chamber as the final layer of the sandwich complex. Finally, the excess secondary antibody solutions were removed using a PBS solution.

### 2.4. Laser Drilling Set-up

**Figure 3.**
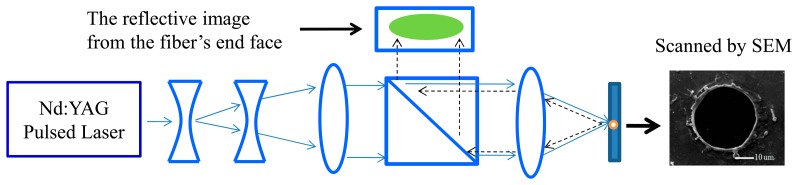
Nd:YAG pulsed laser drilling system was set up to drill a 30 um hole at the center of the proximal end reflector.

A 532-nm Nd:YAG laser and a set of lenses were applied in the laser-drilling system, which was used to drill a 30-μm hole on the end face of the fiber reflector (Figure 3). Both concave lenses were 25.4 mm in diameter and had a focal length of 50 mm, and both convex lenses were 50 mm in diameter with focal lengths of 170 mm (left lens) and 50 mm (right lens). The laser-drilling power was set at 10 mJ. To control the size of the drilling hole, a portion of the collimated laser beam was projected through a beam splitter and onto a screen for monitoring the size of the collimated laser beam throughout the drilling process.

### 2.5. Resonator Fundamentals

**Figure 4.**
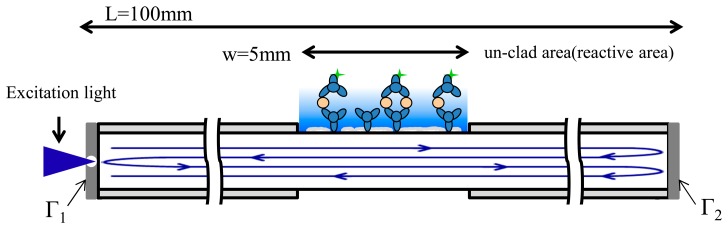
Schematic diagram of the FOBS with a pair of reflectors on the fiber end-face.

The total emission of fluorescence intensity *E_t_* was determined by summing all fluorescence intensity values from the resonant optical fiber biosensor. The equation was obtained by analyzing the propagation and reflection of the coupled light in the optical resonators, as expressed in Equation (1):

(1) Et=I0D11/2D21/2α+I0D13/2D23/2Γα2+I0D15/2D25/2ΓΓ2β1α+I0D17/2D27/2Γ22Γ1βα+I0D19/2D29/2Γ22Γ12β2α+I0D111/2D211/2Γ23Γ12β2α+…=I0D11/2D21/2α×[∑n=0∞βn(D1D2)2n(Γ1Γ2)n+D1D2Γ2×∑n=0∞βn(D1D2)2n(Γ1Γ2)n]=I0D11/2D21/2α×1+D1D2Γ21−β(D1D2)2Γ1Γ2

where *I*_0_ is the excitation light intensity, and α is the fluorescence excitation efficiency. In Figure 4, *D_1_* and *D_2_* are the optical attenuation of the optical fiber outside and inside the reactive region {*D_1_* = exp[−*r_1_(L* − *w)*], *D_2_* = exp(−*r_2_w*), *D_1_^1/2^* = exp[−*r_1_(L* − *w)*/*2*], *D_2_^1/2^* = exp(−*r_2_w/2*); *I_0_D_1_^1/2^D_2_^1/2^α* denotes the intensity of the light at the middle of the fiber; *r*_1_ is the attenuation coefficient of the clad fiber; *r*_2_ represents the attenuation coefficient of the unclad fiber (removed using a CO_2_ laser); *L* represents the fiber length; and *w* denotes the reaction (unclad) length}. The coefficient *β* denotes the effective reflection area of the first reflector (*β* = 1 − laser drilling hole area / fiber core area), while Γ_1_ and Γ_2_ represent the reflectivity of the proximal end (first) and distal end (second) reflector. Therefore, the fluorescence intensity amplification factor of the resonator can be obtained from Equation (2):

(2) $$\text{Amplification}\;\text{Factor}=\frac{1+\left(D_{1}D_{2}\right)\Gamma_{2}}{1-\beta {\left(D_{1}D_{2}\right)}^{2}\Gamma_{1}\Gamma_{2}}$$

## 3. Results and Discussion

### 3.1. Experimental Fluorescence Intensity Amplification Achieved Using the Resonator Structure

**Figure 5.**
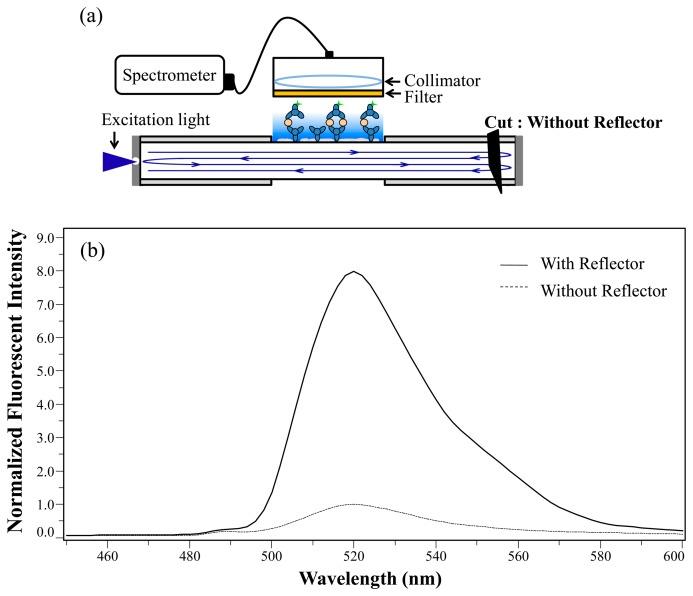
(**a**) Biosensor structure with direct reflective coating of Ag/Al reflector on the end-faces. The second reflector was cut off to demonstrate the termination of the resonance condition “without reflector”; (**b**) Normalized fluorescent intensity spectrum with reflector (solid line) and without reflector (dashed line).

A resonator-type biosensor structure was developed by directly-coating Ag/Al onto the fiber end faces at both ends of the biosensor, as shown in Figure 5a. The coating was formed by a 500-nm-thick Ag layer and a 100-nm-thick Al layer, both of which exhibited high reflectivity (92%). In Figure 5b, the normalized fluorescence spectrum of the biosensor was operated in a resonant condition with the reflector and compared with that in a non-resonance condition and without the reflector (by cutting off the distal end (second) reflector).

### 3.2. Theoretical Fluorescence Intensity Amplification

**Figure 6.**
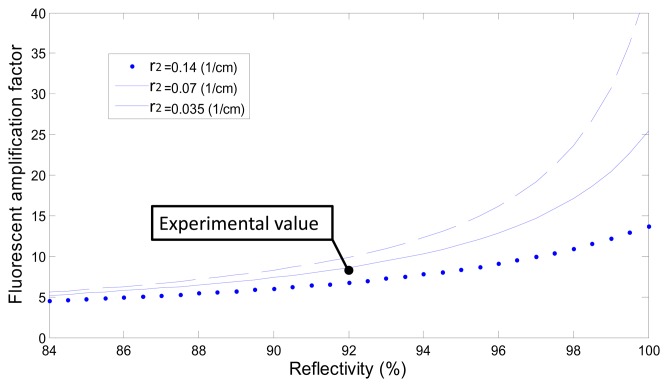
Simulation of the amplification factor *versus* reflectivity and optical attenuations *r_2_* in the reactive area.

According to the measurement results, using the resonant structure and the reflector yielded an amplification factor of eight. In this study, the multimode optical fiber attenuation was 30 dB/km at 488 nm; therefore, it was assumed that the degree of attenuation *D*_2_ of the reactive area (*i.e.*, the unclad area (*D*_2_ in Section 2.5)) varied, as shown in Figure 6. Many parameters affected the amplification factor; for example, if the reflectivity Γ_1_ and Γ_2_ are near 99% (Equation (1) in Section 2.5), then an *r*_2_ value of 0.07 (1/cm) indicates that the optical attenuation is 30,000 dB/km, *L* = 100 mm, *w* = 5 mm, and *β* = 99%. The theoretical amplification factor exceeded 20-fold, as shown in Figure 6, and the effect of optical attenuation in the reactive area, *D*_2_ = exp(−*r_2_w*), influenced the amplification factor. Therefore, *r*_2_ was affected by several factors, such as the degree of cladding removal, the presence of residual impurities on the surface of the reactive region, and the absorption of laser energy from the fluorescent molecules. To further test the influence of the optical attenuation *r*_2_, various values were applied (0.14, 0.07, and 0.035).

The simulation results shown in Figure 6 indicate that further amplification was limited by the optical attenuation *r*_2_ in the reactive region; specifically, when *r*_2_ was low, additional fluorescence amplification was observed. Thus, completely removing the cladding and residual impurities was critical for amplifying the fluorescence.

**Figure 7.**
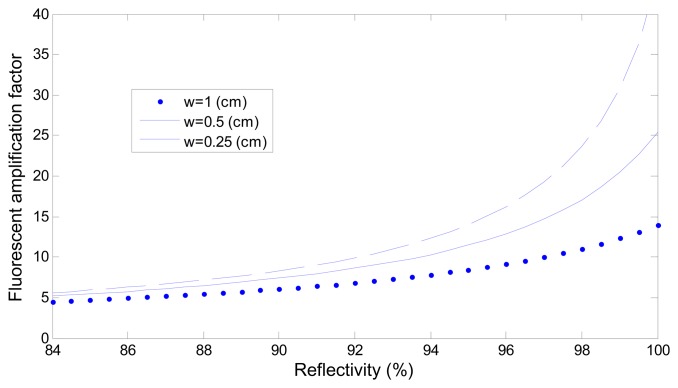
Simulation of the amplification factor *versus* reflectivity and lengths *w*, *r_2_* = 0.07 (1/cm) at the reactive area.

Figure 7 depicts the effect of adjusting the reactive region length *w*. The figure shows that greater amplification was achieved when the reactive region length *w* was shorter, implying that the more the reaction area is shortened to reduce the laser energy loss, the greater the obtainable amplification is. However, reducing the length of the reaction area caused a reduction in the collection of the fluorescence signal intensity; therefore, using a reaction area of an appropriate length generates stronger fluorescence signals.

### 3.3. Other Applications of the Resonator Structure

When the excitation light was perfectly focused on the drilling hole of the end face of the fiber, the fiber was fully used to enhance the power of the excitation light source. Furthermore, because this structure requires a low-power laser, the cost of fabrication was reduced, thereby facilitating the miniaturization of the biosensor.

Resonators are typically used to enhance the intensity of signals, but they have rarely been used in optical fiber biosensors. To enhance the sensitivity of evanescent wave FOBSs, this study developed a novel resonator structure [23]. The structure can be used to enhance evanescent waves for fiber-surface-enhanced Raman scattering (SERS) [24] by enhancing the evanescent wave energy, thereby further increasing the SERS signal. The resonator structure could also be used to amplify evanescent waves for fluorescence-enhancements with metallic nanoparticles in surface-plasmon-resonance (SPR) applications [25]. The resonator structure and the fiber SPR structure can be existed simultaneously. The resonator structure can amplify the excitation light and the metallic nanoparticles can enhance the fluorescence. The combination of the resonator and the fiber SPR biosensors structure can generate a greater fluorescent signal.

## 4. Conclusions

This study aimed to improve the efficiency of evanescent wave excitation. Using an optical resonator structure enabled the excitation light to be fully used in the optical fiber biosensor. The proposed resonator structure is a fundamental approach to improve the signal intensity of evanescent wave FOBSs.

This paper reveals that the resonator structure (with 92% reflectivity reflectors) amplified the fluorescence intensity by a factor of eight. Theoretically, the fluorescence intensity could be amplified by a factor of 20 if the reflectivity were exceeded 98%. The proposed approach offers a practical and inexpensive method for efficiently improving the sensitivity of evanescent wave FOBS, and thus has considerable potential in various optical applications, including optical fiber biosensing, the enhancement of fluorescence, and Raman spectroscopy.
